# An Outbreak of Tuberculosis among Cattle at an Altitude of 7400 Feet

**Published:** 1900-07

**Authors:** S. W. McClure

**Affiliations:** Helena, Montana


### AN OUTBREAK OF TUBERCULOSIS AMONG CATTLE AT AN ALTITUDE OF 7400 FEET.

By S. W. McClure, V.M.D.,
HELENA, MONTANA.

While in the performance of my duties as deputy State veterinarian of Montana, I was ordered by Dr. M. E. Knowles to investigate the origin and extent of an outbreak of tuberculosis on the ranch of J. H. Baker and A. M. Davidson, of Madison County, Montana. The location of the ranch was such as to favor the cure of tuberculosis rather than its rapid spread and extreme fatality. The situation of this ranch was peculiar, and it seems strange that this extremely healthful situation was the first to give Montana a well-defined outbreak of tuberculosis.

The ranch comprises 4000 acres of level buffalo-grass range; its altitude, according to the geological survey, is 7400 feet, and the range itself is situated at the foot of a chain of mountains, the highest point of which is 11,334 feet above the sea. There being no fence along the bottom of these mountains, the cattle feed up the sides as far as it is possible to go, sometimes reaching the altitude of 9000 feet. The ranch is watered by a beautiful mountain stream, and about a mile from the base of the mountain there is a large body of hot alkaline water, of which I frequently saw the cattle drink.

This outbreak affords a good opportunity for a study of the contagiousness of tuberculosis, as Mr. Baker has been on this ranch for about twelve years, during which time he had made few changes in his original stock, which was composed of apparently healthy range-cattle. Five years previous to the time of my visit he had purchased a Jersey bull at a high price from an Eastern breeder. After having the bull in contact for about a year with his other cattle, numbering 150 head, he noticed that the bull had developed an enlargement of his jaws, that he made a loud noise in breathing and coughed considerably. The bull gradually became very thin, and Mr. Baker states he choked to death about one year after he was first noticed affected. An incision into the enlargement at his throat showed it to contain a large quantity of thick pus. Within the last two months, three other cattle have died after showing the same outward symptoms.

At the time of my visit, in April, I rode through the herd and picked out five cattle as probably tuberculous. I immediately killed and held postmortem examinations on the five condemned animals and found them to be in an advanced tuberculous condition, many of the glands having undergone cheesy and calcareous changes. The anatomical parts involved were as follows: postpharyngeal lymphatic glands, mediastinal glands, lumbar lymphatic glands, axillary lymphatic glands, mesenteric lymphatic glands. There were tubercular patches in the liver and, in one instance, tuberculosis of the meninges. The animals were all very much emaciated, and would have survived only a few months at the longest. The peculiar point in connection with the necropsy was the remarkable freedom of the organs of the thoracic cavity from any tubercular lesions, as only in one animal did I find a small, calcified, well-encapsulated tubercular area in the lungs. Three of the animals suffered from tubercular arthritis.

I report this case to show that tuberculosis may spread under the best sanitary conditions possible. The truth of this statement is seen when it is understood that there was no other possible source from which the infection could have occurred, except the said Jersey bull, and as the cases all appeared in three- and four-year old cattle, and as these cattle were on the range with the bull at the time he was noticed to be tuberculous, and as the cattle were never stabled, never fed from troughs or watered from any place but the mountain streams and hot alkaline springs, it shows that tuberculosis will spread under the best climatic conditions possible.

The cattle under consideration had never seen a stable, and had always been kept on the range excepting during a few very stormy days when they went into a little grove of pine-trees for shelter. There is also a log corral about ten feet high into which the cattle sometimes go when it is windy. The spaces between the logs of this corral are not chinked and it is covered with pine-boughs and straw. The cattle are all high-grade short-horns, except the Jersey bull that died and fifteen or twenty of his get. The cattle affected were not by the Jersey bull. The Jersey bull had been purchased for the purpose of raising a few cows for dairy purposes, and was not turned in with the general herd until the breeding season was over.

The absence of any lesion in the lungs was possibly due to the following reasons: The breathing of air free from dust and infectious microbes, the increased oxidation of blood and tissue from sunlight, cold air, and reduced pressure found at these altitudes; the increased quantity of blood circulating in the lungs caused by the reduced pressure, the freedom of circulation being aided by the increased chest movements seen in high altitudes.

It would be interesting to determine the extent to which tuberculosis is now prevailing in this herd, but as the application of the tuberculin-test is impossible under range conditions, we shall have to be content with a physical examination. Three other cattle show enlarged postpharyngeal glands and are now in quarantine.

The postmortem notes follow:

Case I.—Red heifer, aged two and a half years, much emaciated; she had just given birth to a dead fetus; had been noticed sick for three months. Owner had noticed enlargement of the postpharyngeal lymphatic glands for about the same length of time. Killed by shooting and then bled. Postpharyngeal glands much enlarged, cheesy and calcareous. In the centre of the gland about a cupful of creamy, tuberculous pus. Mediastinal glands in an advanced state of calcareous degeneration, but no pus present. Tuberculous area in the caudal lobe of each lung. The area was calcareous and cheesy and firmly encapsulated, and had no apparent blood-supply. Mesenteric lymphatic glands enlarged and cheesy. Costal pleura contained some few tubercles. The axillary glands and also the lymphatic glands of hock were tuberculous and much enlarged. Diagnosis, tuberculosis.

Case II.—Red steer, aged two and a half years, very much emaciated. Would stand for ten or fifteen minutes at a time and turn in a circle always to the left side, then get his head up into a corner and remain there until disturbed; always carried his head low and to the left side. Shot and then bled. Small tuberculous area in the caudal lobe of the left lung; mesenteric glands enlarged and œdematous, showing much evidence of inflammation; glands of the shoulder and hock tuberculous; calcified bodies between the skin and the inferior surface of the sternum, about the size of a large pinhead. The liver was small and atrophied. The gall-bladder distended. I think that this steer suffered from meningeal tuberculosis, but on account of being shot twice through the brain, I could find no trace of the lesion, perhaps, on account of the hemorrhage. Diagnosis, tuberculosis.

Case III.—Roan cow, three and a half years old, and in fine condition; sucking calf three weeks old. Shot and then bled. Postpharyngeal glands much enlarged; on incision revealed a cavity filled with pus. Tuberculous patches on the posterior surface of the diaphragm; tubercular nodules in the wall of the intestine. All other glands much enlarged and œdematous, showing evidence of chronic inflammation, but no cheesy tubercles present. Diagnosis, tuberculosis.

Case IV.—Black steer; emaciated. Shot and then bled. Postpharyngeal glands much enlarged; mediastinal and lumbar glands cheesy and calcified. Periosteum of humerus and of the knee- and hock-joints contained red ecchymotic spots, but no tuberculous ulcers apparent. This steer was very stiff in right front-leg and left hind-leg. Diagnosis, tuberculosis,

Case V.—Roan cow, aged four years. Postpharyngeal lymphatic glands much enlarged and contained tubercular abscess. Mediastinal glands; cheesy and calcified lungs normal; lumbar glands and mesenteric lymphatic glands tuberculous; tuberculous patches on the posterior surface of the diaphragm, and three tuberculous abscesses in the liver. Diagnosis, tuberculosis.

---

Heir-at-Law, 2.05$\frac{3}{4}$, not long ago broke his leg while at play in his paddock. The fracture was a clean one, but the resident veterinary surgeon at the Village Farm set the bones; the horse was put in a sling and chances were taken on their knitting. This they have done to such good purpose and effect that Heir-at-Law is once again going around sound, and if one did not know that he had had his leg broken the fact would never be suspected from his appearance and gait.

When domestic animals have the opportunity to make a change of food they do so. This may be noticed when they are on the pasture, some being content with certain grasses, while others will seek some other kind. They also prefer a change at times in the winter, especially when they receive no food of a succulent character Provide ensilage, turnips, carrots, or any other kind of food other than dry grain and hay.

